# Topsy-Turvy: integrating a global view into sequence-based PPI prediction

**DOI:** 10.1093/bioinformatics/btac258

**Published:** 2022-06-27

**Authors:** Rohit Singh, Kapil Devkota, Samuel Sledzieski, Bonnie Berger, Lenore Cowen

**Affiliations:** Computer Science and Artificial Intelligence Lab., Massachusetts Institute of Technology, Cambridge, MA 02139, USA; Department of Computer Science, Tufts University, Medford, MA 02155, USA; Computer Science and Artificial Intelligence Lab., Massachusetts Institute of Technology, Cambridge, MA 02139, USA; Department of Mathematics, Massachusetts Institute of Technology, Cambridge, MA 02139, USA; MIT Schwarzman College of Computing Cambridge, MA 02139, USA; Department of Computer Science, Tufts University, Medford, MA 02155, USA

## Abstract

**Summary:**

Computational methods to predict protein–protein interaction (PPI) typically segregate into sequence-based ‘bottom-up’ methods that infer properties from the characteristics of the individual protein sequences, or global ‘top-down’ methods that infer properties from the pattern of already known PPIs in the species of interest. However, a way to incorporate top-down insights into sequence-based bottom-up PPI prediction methods has been elusive. We thus introduce Topsy-Turvy, a method that newly synthesizes both views in a sequence-based, multi-scale, deep-learning model for PPI prediction. While Topsy-Turvy makes predictions using only sequence data, during the training phase it takes a transfer-learning approach by incorporating patterns from both global and molecular-level views of protein interaction. In a cross-species context, we show it achieves state-of-the-art performance, offering the ability to perform genome-scale, interpretable PPI prediction for non-model organisms with no existing experimental PPI data. In species with available experimental PPI data, we further present a Topsy-Turvy hybrid (TT-Hybrid) model which integrates Topsy-Turvy with a purely network-based model for link prediction that provides information about species-specific network rewiring. TT-Hybrid makes accurate predictions for both well- and sparsely-characterized proteins, outperforming both its constituent components as well as other state-of-the-art PPI prediction methods. Furthermore, running Topsy-Turvy and TT-Hybrid screens is feasible for whole genomes, and thus these methods scale to settings where other methods (e.g. AlphaFold-Multimer) might be infeasible. The generalizability, accuracy and genome-level scalability of Topsy-Turvy and TT-Hybrid unlocks a more comprehensive map of protein interaction and organization in both model and non-model organisms.

**Availability and implementation:**

https://topsyturvy.csail.mit.edu.

**Supplementary information:**

Supplementary data are available at *Bioinformatics* online.

## 1 Introduction

We focus on the problem of predicting PPIs from sequence data without the computational expense of multiple sequence alignments, thus enabling genome-scale predictions. Classically, the physical protein–protein interaction (PPI) prediction problem has been studied in two settings: one, where we only have access to each protein’s amino acid sequence and must determine from the sequence data alone if the two proteins bind (e.g. Chen *et al.*, 2019; Hashemifar *et al.*, 2018; Sledzieski *et al.*, 2021; Zhang *et al.*, 2012). The other infers new interactions from the global topological properties of known PPI connections using either a simple rule such as ‘proteins with many common interaction partners are likely to also interact’, or more sophisticated diffusion-based network embeddings (e.g. Coşkun and Koyutürk, 2021; Cowen *et al.*, 2017; Devkota *et al.*, 2020; Hamilton *et al.*, 2018; Huang *et al.*, 2020; Kovács *et al.*, 2019; Yuen and Jansson, 2020).

Our previous work introduced D-SCRIPT (Sledzieski *et al.*, 2021), a structure-aware deep-learning model for predicting protein interactions. D-SCRIPT takes a bottom-up view, learning about protein interactions pair-by-pair through the lens of (inferred) protein structure and, by leveraging a natural language based protein sequence representation, was shown to achieve state-of-the-art cross-species generalizability. While we originally trained D-SCRIPT on pairwise human PPI data, we pursue here the intuition that the wealth of network-level global information available could potentially improve predictive performance if integrated during the training phase. Unfortunately, we found scant guidance in the literature for how to make use of both types of information simultaneously: existing PPI prediction methods (such as those listed above) either take exclusively a top-down or bottom-up approach, ignoring the other approach entirely.

Here, we propose a new approach, **Topsy-Turvy**, that integrates graph-theoretic (top-down) and sequence-based (bottom-up) approaches to PPI prediction in the training phase of our sequence-based predictor. Topsy-Turvy introduces a multi-objective training framework that takes a pair of protein sequences as input, with the supervision provided by *both* experimentally determined PPIs (in the same manner as D-SCRIPT), as well as with global topological measures of protein pair compatibility. Importantly, it only requires protein sequences as inputs when making predictions—network information is used only during training. Since the trained Topsy-Turvy model makes predictions using just sequence data, it is particularly valuable in non-model organisms where almost no PPI data is available (Sledzieski *et al.*, 2021). We also investigate whether AlphaFold-Multimer (Evans *et al.*, 2021), a very recent method for protein-complex structure prediction, can instead be adapted to solve our PPI prediction task; however, we found it to be 100 000 times slower than Topsy-Turvy. Due to its computational efficiency, Topsy-Turvy is applicable in genome-wide prediction settings where AlphaFold-Multimer would be infeasible.

While Topsy-Turvy requires no pre-existing experimental data in the species of interest, for cases where some such data *is* available (e.g. in worm or fly) we devise a hybrid model, **TT-Hybrid**, that is able to take advantage of species-specific network data. TT-Hybrid embodies a principled approach to combining the Topsy-Turvy sequence scores with GLIDE (Devkota *et al.*, 2020) scores to make PPI predictions; we chose GLIDE after benchmarking it against the widely used node2vec (Grover and Leskovec, 2016) (Section 3.1). We show that TT-Hybrid performs better than its competitors, or just Topsy-Turvy or GLIDE alone.

This work has several key conceptual advances—(i) whereas the D-SCRIPT algorithm showed that informative features generated by a protein language model enable transfer learning of the structural basis of interaction, we show that we can likewise transfer global patterns of PPI organization by integrating a topological compatibility score into the loss function. (ii) We approach the synthesis of bottom-up and top-down approaches as a multi-objective training problem that balances between structural and topological considerations when predicting PPIs. Except for the recent work of Yang *et al.* (2020), such integrative approaches in prior work have been rare. (iii) We provide a framework for accurately predicting PPIs in a variety of settings—both cross-species, where no training data is available in the target species, as well as in species that have limited experimentally determined PPIs.

In a cross-species setting, Topsy-Turvy achieves state-of-the-art results, substantially improving upon the cross-species generalizability of PIPR (Chen *et al.*, 2019), a deep learning method by Richoux *et al.* (2019) and D-SCRIPT. We investigate Topsy-Turvy’s improved performance, finding that it performs better not only on interactions involving hub nodes in the target species but even more so on low-degree nodes; this suggests that the measured outperformance is not simply due to ascertainment bias (Carter *et al.*, 2013) (Sections 3.3 and 3.4). We also investigated Topsy-Turvy’s usefulness in settings where sufficient PPI data exists so that a putative interaction between two proteins *could* also be predicted using global methods. We show that TT-Hybrid’s principled synthesis of the scores from the network-based GLIDE method (Devkota *et al.*, 2020) and Topsy-Turvy yields state-of-the-art performance in this setting as well.

## 2 Materials and methods

### 2.1 Overview of Topsy-Turvy

**Fig. 1.**
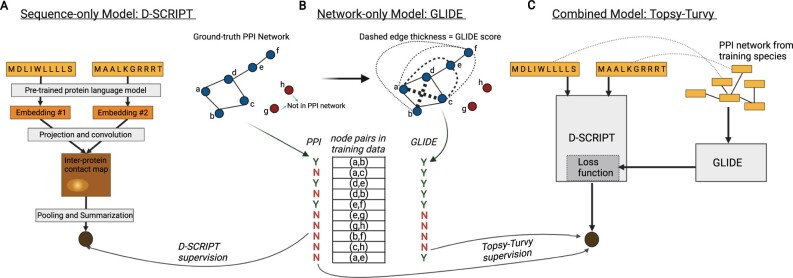
Topsy-Turvy synthesizes sequence-to-structure-based prediction using D-SCRIPT with network-based prediction using GLIDE. (**A**) D-SCRIPT uses a protein language model to generate representative embeddings of protein sequences, which are combined with a convolutional neural network to predict protein interaction. It is supervised using binary interaction labels from the training network and regularized by a measure of contact map sparsity. (**B**) GLIDE scores all possible edges using a weighted combination of global and local network scores which are learned from the edges already in the training network. (**C**) Topsy-Turvy is supervised with both the binary interaction labels of the true (training) network and with the GLIDE predicted scores, thus integrating bottom-up and top-down approaches for PPI prediction into the learned Topsy-Turvy model

Topsy-Turvy provides a general paradigm to integrate a bottom-up sequence-based and top-down global method: for these two components in Topsy-Turvy we choose D-SCRIPT for the sequence-based prediction, and GLIDE for the network-base prediction. We next briefly review D-SCRIPT and GLIDE. In Topsy-Turvy, we adapt the D-SCRIPT model to synthesize the two by adding to it a network-dependent loss term inferred from the GLIDE model (Fig. 1).

### 2.2 Background: sequence-based prediction with D-SCRIPT

To make bottom-up, structure-aware predictions of PPIs, we use D-SCRIPT, a state of the art method for sequence-based PPI prediction across species. Briefly, D-SCRIPT operates in two stages. First, we generate a feature-rich representation of each protein using a protein language model (PLM) (Bepler and Berger, 2019, 2021); next, these features are combined using a convolutional neural network to predict interaction. The Bepler & Berger PLM was chosen to extract structurally relevant features. Leveraging it, the D-SCRIPT architecture mimics the structural mechanism of protein interaction and includes an intermediate representation that encodes the intra-protein contact map. During inference, these predicted contact maps were shown to substantially recapitulate ground-truth binding mechanisms despite no structure-based supervision or inputs. To achieve this, the training procedure for D-SCRIPT minimizes a hybrid loss that contains terms measuring both the binary cross-entropy of predictions $\left(L^{\mathrm{BCE}}\right)$ and the overall magnitude of the contact map $\left(L^{\mathrm{MAG}}\right)$ which enables sparse and realistic contact map prediction. The relative weight of these loss terms are balanced by a hyperparameter *λ*. We emphasize that D-SCRIPT requires only the amino acid sequence of a protein pair to make predictions.

### 2.3 Background: network-based prediction with GLIDE

To make top-down, network-based predictions of PPIs in a species, we use GLIDE (Devkota *et al.*, 2020), a state-of-the-art method that combines local (neighborhood-based) and global (spectral) graph-theoretic techniques for quantifying the likelihood of an interaction between every protein-pair in the network. As part of our initial explorations, we also evaluated node2vec (Grover and Leskovec, 2016), another spectral approach for link prediction. However, we found GLIDE to outperform node2vec substantially on the PPI link prediction task (Section 3.1) and hence chose it as the link prediction technique in this article. GLIDE combines a simple local score that captures shared-neighbor relationships in the dense core with a diffusion-based embedding that encapsulates the network structure in the periphery. While local metrics accurately capture the likelihood of links between proteins in the same local neighborhood, their performance drops significantly as the distance between proteins increases. The opposite is true for global metrics.

GLIDE incorporates both local and global metrics into a single score in such a way that each metric is leveraged in the region of the network where it is most accurate. We use Common Weighted Normalized (CWN) as our local metric, and the inverse of the Diffusion State Distance (${\text{UDSED}}^{\gamma }$) as our global metric while computing the GLIDE score. For a more detailed description of CWN and ${\text{UDSED}}^{\gamma }$ metrics, see the Supplementary Appendix S1.1.

Following Devkota *et al.* (2020), we compute the aggregate GLIDE score between each pair of nodes as:

(1) $$\mathrm{GLIDE}(p,q)=\text{exp}\;\left(\frac{\alpha \cdot u(p,q)}{u(p,q)+\beta }\right)CWN(p,q)\;+u(p,q)$$

where (*p*, *q*) is a candidate protein pair and $u(p,q)=1/{\text{UDSED}}^{\gamma }(p,q)$. We chose the default values of *α* and *β* as suggested by Devkota *et al.* (2020) (α=0.1,β=1000). These choices make the local embedding dominant, whenever available, with the global embedding being used to break ties and order nodes with the same local score. For the CWN local score, node-pairs with no common neighbors will have CWN(p,q)=0 and only the global *u* term will be used.

### 2.4 Network-dependent loss term

Topsy-Turvy retains the protein language model feature generation and convolutional neural net architecture of D-SCRIPT, with changes made to the training approach and loss function. To synthesize this model with link-based prediction, we introduce the additional task of predicting GLIDE scores between proteins, formulating it as an extra loss term in the objective. The entire model is then trained end-to-end.

In the original D-SCRIPT model, the loss function was a weighted sum, $L=\lambda L^{\mathrm{BCE}}+(1-\lambda )L^{\mathrm{MAG}}$, that combined the binary cross-entropy (BCE; Sledzieski *et al.*, 2021) loss with a regularization penalty related to the contact map’s magnitude. To incorporate a network term, we add a sub-objective to the classification component:

(2) $$L=\lambda (L^{\mathrm{BCE}}+g_{p}L^{\mathrm{GLIDE}})+(1-\lambda )L^{\mathrm{MAG}}$$

where *L^GLIDE^* represents the loss when predicting GLIDE estimates and $0\leq g_{p}\leq 1$ is a hyperparameter indicating its relative importance (at *g_p_* = 0, the function reduces to the original D-SCRIPT loss). To compute *L^GLIDE^*, we first generate GLIDE scores for every negative training example by computing the component CWN and ${\text{UDSED}}^{\gamma }$ scores on the PPI network defined by the positive examples in the training set. For a protein pair (*p*, *q*), the loss *L^GLIDE^* is defined as

(3) $$L^{\mathrm{GLIDE}}(p,q;\;g_{t})=\text{BCE}(\;y(p,q),\;\;1_{\mathrm{GLIDE}(p,q)\geq g_{t}})$$

where $g_{t}>0$ is a hyperparameter, *y*(*p*, *q*) is Topsy-Turvy’s predicted score for the protein pair (*p*, *q*). 1 is the indicator function corresponding to the predicate $\mathrm{GLIDE}(p,q)\geq g_{t}$. This formulation corresponds to binarizing GLIDE scores at the score threshold *g_t_* and then applying the standard BCE loss. For convenience, we define *g_t_* in terms of a percentile cutoff on the distribution of *GLIDE*(*p*, *q*) scores (i.e. $0<g_{t}<100$), rather than directly as a numeric threshold.

In formulating *L^GLIDE^*, we chose to binarize GLIDE scores and compute a BCE loss, rather than keeping continuous-valued GLIDE scores and using a different functional form for the loss. Doing so allowed us to mimic the form of the existing BCE-based loss, letting us calibrate the relative weights of *L^BCE^* and *L^GLIDE^* simply by *g_p_*. Using GLIDE’s continuous scores would have made this calibration difficult, since the un-normalized GLIDE scores are unevely distributed (for the human PPI training network: minimum = 0, median = 0.31; 75th-percentile = 0.40; maximum = 2.71) and do not follow a convenient closed form.

The addition of the GLIDE loss term to the model training accounts for the observation that the original D-SCRIPT loss measures only pairwise interaction, and is unaware of global network structure. Since the GLIDE score of a protein pair takes into account local and global network properties, the GLIDE component of the loss should incorporate network-wide information into the predictions. Specifically, since D-SCRIPT prioritizes precision and is more likely to miss true interacting pairs than GLIDE, the absence of strong structural evidence of interaction could be supplemented by strong network evidence.

### 2.5 TT-Hybrid

During inference, Topsy-Turvy requires only protein sequences as input. When making predictions in a species where some PPI data is also available, predictions from pre-trained Topsy-Turvy (trained on data from another species) can be combined with GLIDE predictions informed by the target species’ PPI network. We note that these GLIDE scores are distinct from those corresponding to the training species; the latter were used only during training. To take advantage of the PPI network in the target species when available, we designed TT-Hybrid that can be applied on query protein-pairs where both GLIDE and Topsy-Turvy scores are available. We note that this requires both proteins of the queried pair to be present in the target species’ PPI network; otherwise, only Topsy-Turvy can be used. TT-Hybrid computes a weighted sum of Topsy-Turvy and GLIDE predictions for a query protein-pair, with the score for a protein pair (*p*, *q*) being:

(4) TT-Hybrid(p,q)=1·GLIDE(p,q)+w·Topsy-Turvy(p,q)

For simplicity, we have set the weight of GLIDE scores to 1, since only the relative weighting of the two scores matters. In this article, we trained Topsy-Turvy on human PPI data and have evaluated it on other species. During the training phase, we held out some human PPI data for validation. We calibrated *w* on this held-out human data using logistic regression.

We started by selecting protein pairs corresponding to the edges of the held-out human PPI subnetwork (see Section 3.2 for dataset details). These pairs were labeled positive; negatively labeled pairs corresponded to random pairs of proteins from the subnetwork. The ratio of negative to positive examples was set to 10:1 to account for the inherent class imbalance in PPI data (see Section 3.2 for discussion). To avoid bias arising from data leakage, we also required that none of the examples occur in the original training data for Topsy-Turvy. We computed GLIDE and Topsy-Turvy scores for each protein pair, these methods having previously been trained on the rest of human PPI data. We then fitted a logistic regression model that sought to predict the label of a protein pair using its GLIDE and Topsy-Turvy score. The TT-Hybrid calibration weight *w* is chosen as the ratio of logistic regression coefficients, $c_{\text{Topsy-Turvy}}/c_{\text{GLIDE}}$. Our computation yielded *w *=* *0.3268, and we recommend the use of this value when applying TT-Hybrid in other species, as is done in the results presented here. If enough PPI data is available in the target species that a portion of it can be set aside, the held-out portion can be used to calibrate *w* specifically for the target species. To avoid the risk of data leakage, however, the same set of PPIs should not be used to both calibrate *w* and compute the GLIDE score inputs to TT-Hybrid.

### 2.6 Hyperparameter selection and model training

The hyperparameters *g_p_* (the relative weight of GLIDE versus binary cross-entropy loss) and *g_t_* (the binarization threshold for GLIDE scores) play a crucial role in Topsy-Turvy and we sought to estimate them from cross-validation runs on the human PPI dataset. We note that all Topsy-Turvy and TT-Hybrid results presented in this article are from models trained on human data but evaluated on out-of-sample, non-human data. To perform the hyperparameter search, we did cross-validation runs on the *entire* human PPI network, since GLIDE scores computed on smaller subnetworks might not be representative of the full network’s characteristics. Due to the computational expense of such runs, however, we modified the standard grid-search approach. Initial, small scale explorations suggested *g_t_* = 90 to be a promising choice. We first performed a grid search on *g_p_*, fixing *g_t_* to 90. This yielded $g_{p}=0.2$ as the suggested choice (Table 1a) and we then performed a grid search for *g_t_*, with *g_p_* fixed to this choice. The second search indicated $g_{t}=92.5$ to be the best choice (Table 1b), and we accordingly chose $g_{p}=0.2,\;g_{t}=92.5$ as the hyperparameter settings for Topsy-Turvy training.

**Table 1.** Hyperparameter search: cross-validation AUPR (area under precision–recall curve) scores on full human PPI network for (a) grid search for *g_p_*, with *g_t_* fixed to 90 (estimated from small-scale explorations), (b) grid search for *g_t_*, with *g_p_* fixed to 0.2 [i.e. the optimal value from (a)]

**(a)**	
g_p_ (with g*_t_* = 90)	AUPR
0.1	0.739
**0.2**	**0.802**
0.4	0.759
0.8	0.760
**(b)**	
*g_t_* (with g*_p_* = 0.2)	AUPR
90	0.697
**92.5**	**0.824**
95	0.691
97.5	0.690

*Note*: The metrics reported in the tables are the validation AUPR scores maximized over three epochs of training.

#### 2.6.1 Additional implementation details

We implemented Topsy-Turvy in PyTorch 1.2.0 and trained with a NVIDIA Tesla V100 with 32 GB of memory. Embeddings from the pre-trained Bepler and Berger model were produced by concatenating the final values of the output and all hidden layers. Apart from these pre-trained embeddings, Topsy-Turvy was trained end-to-end and did not use pre-trained D-SCRIPT model weights. However, we used the same hyperparameters as in Sledzieski *et al.* (2021) for the relevant components of our model’s architecture: a projection dimension of *d *=* *100, a hidden dimension of *h *=* *50, a convolutional filter with width 2w+1=7, and a local max-pooling width of *l *=* *9. Furthermore, we used λ=0.05 for calculating the training loss, choosing it based on early, small-scale explorations. Weights were initialized using PyTorch defaults. Model training parameters were set within ranges commonly used in deep learning literature: we used a batch size of 25, the Adam optimizer with a learning rate of 0.001, and trained all models for 10 epochs.

## 3 Results

We start by presenting a comparative assessment of GLIDE and node2vec for PPI link prediction; the results of this analysis motivated our choice of GLIDE as the network-theoretic component of the Topsy-Turvy model. We next evaluate the cross-species generalizability of Topsy-Turvy, showing how incorporating network data during training results in superior performance in other species, using only sequence data for prediction. We note that in the typical cross-species setting, purely network-based methods like GLIDE are not applicable since they can only make predictions for pairs where both proteins exist in the training PPI network and hence cannot be applied to out-of-sample proteins. We therefore evaluated Topsy-Turvy against methods that require only sequence-based inputs (like D-SCRIPT), assessing if co-supervising Topsy-Turvy with topological information allows it to learn aspects of protein interaction that carry across species. As we show, it does, and in subsequent analyses we investigate various aspects of the comparison more deeply, also addressing the issue of ascertainment bias in the evaluation network. Lastly, we study how to best apply Topsy-Turvy in instances where PPI data *is* available and GLIDE would be applicable directly. We find that while GLIDE is broadly informative about the species-specific network rewiring, better performance can be achieved by TT-Hybrid, a combination of Topsy-Turvy and GLIDE.

### 3.1 Comparison of GLIDE and node2vec

In our initial explorations, we sought to identify the most appropriate top-down PPI link prediction technique. Toward this, we compared GLIDE to node2vec (Grover and Leskovec, 2016). The node2vec algorithm, also a spectral approach, uses a biased random walk procedure to construct low-dimensional node embeddings. Following the original study, we trained a logistic regression classifier on the Hadamard product of the node embeddings to predict the existence of a link given two candidate proteins. We compared the two methods on the *Drosophila* BioGRID network consisting of 3093 nodes and 25 427 edges. A certain fraction 1−p of the edges were removed from the network (while protecting a random spanning tree to ensure connectivity), and the remaining subnetwork was used to train the node2vec and the GLIDE models. The removed edges were then used as positive test examples for evaluation. For negative examples, we randomly sampled 254 270 node-pairs (or 10 times the positive edge count) that were not present in the original network. The negative examples, like the positive edges, were also separated into train and test sets using the same parameter *p*. The dimension of the node2vec embedding was set to 300, i.e. approximately 10% of the node count [following Cho *et al.* (2016); this is also higher than the minimum value of 100, as prescribed by Grover et al.]. We evaluated both node2vec and GLIDE for different values of *p* (which correspond to varying levels of network sparsity), finding that GLIDE outperformed node2vec consistently (Table 2).

**Table 2.** GLIDE and node2vec comparison: AUPR scores for PPI prediction on the *Drosophila* BioGRID network

*p*	GLIDE	node2vec
0.8	**0.737**	0.681
0.6	**0.818**	0.721
0.4	**0.839**	0.664
0.2	**0.805**	0.574

*Note*: Higher values of *p* correspond to a higher proportion of edges preserved in the training network. Bold entries represent best performance.

### 3.2 Integrating network-level information improves predictive performance

*Datasets*: We trained Topsy-Turvy on human PPI data and evaluated it on *Mus* *musculus*, *Drosophila* *melanogaster*, *Caenorhabditis* *elegans*, *Saccharomyces* *cerevisiae* and *Escherichia* *coli*. The dataset selection and pre-processing follows Sledzieski *et al.* (2021): we sourced positive examples from the STRING database (v11) (Szklarczyk *et al.*, 2021), selecting only physical binding interactions associated with a positive experimental-evidence score. Our human PPI set consists of 47 932 positive and 479 320 negative protein interactions, of which we set apart 80% (38 345) for training and 20% (9587) for validation (see Supplementary Appendix S1.2 for details). For each of 5 model organisms (Table 3) we selected 5000 positive interactions and 50 000 negative interactions using this procedure, with the exception of *E.coli* (2000/20 000) where the available set of positive examples in STRING was limited. Each model was trained three times, with different random seeds, and we evaluated the average performance across these runs. We emphasize that Topsy-Turvy is trained end-to-end and does not use a pretrained D-SCRIPT sub-component. For benchmarking, a separate D-SCRIPT model was trained and evaluated identically.

**Table 3.** Topsy-Turvy improves upon D-SCRIPT (Sledzieski *et al.*, 2021), PIPR (Chen *et al.*, 2019) and DeepPPI (Richoux *et al.*, 2019) for cross-species PPI prediction

Species	Model	AUPR	AUROC	FPR	
				0.1 Recall	0.5 Recall
*M.musculus*	PIPR	0.526	0.839	0.002	0.057
	DeepPPI	0.518	0.816	**0.0002**	0.059
	D-SCRIPT	0.663 ± 0.05	0.901 ± 0.02	0.002	0.014
	Topsy-Turvy	**0.735** ± **0.03**	**0.934** ± **0.01**	0.001	**0.009**
*D.melanogaster*	PIPR	0.278	0.728	0.007	0.197
	DeepPPI	0.231	0.659	0.012	0.274
	D-SCRIPT	0.605 ± 0.06	0.890 ± 0.02	0.003	0.022
	Topsy-Turvy	**0.713** ± **0.05**	**0.921** ± **0.02**	**0.001**	**0.011**
*C.elegans*	PIPR	0.346	0.757	0.002	0.148
	DeepPPI	0.252	0.671	0.007	0.252
	D-SCRIPT	0.550 ± 0.08	0.853 ± 0.04	0.003	0.032
	Topsy-Turvy	**0.700** ± **0.04**	**0.906** ± **0.03**	**0.001**	**0.011**
*S.cerevisiae*	PIPR	0.230	0.718	0.017	0.213
	DeepPPI	0.201	0.652	0.018	0.288
	D-SCRIPT	0.399 ± 0.09	0.790 ± 0.06	0.005	0.089
	Topsy-Turvy	**0.534** ± **0.01**	**0.850** ± **0.02**	**0.002**	**0.038**
*E.coli*	PIPR	0.271	0.675	0.005	0.246
	DeepPPI	0.271	0.688	0.004	0.243
	D-SCRIPT	0.513 ± 0.09	0.770 ± 0.03	0.002	0.040
	Topsy-Turvy	**0.556** ± **0.09**	**0.805** ± **0.07**	**0.001**	**0.038**

*Note*: All species were evaluated using models trained on a large corpus of human PPIs. For D-SCRIPT and Topsy-Turvy, we report the average and standard deviation of results from three random initializations. For PIPR and DeepPPI, we report here the results from the study in Sledzieski *et al.* (2021) where the same evaluation scheme and data was used. For all datasets, there is a 1:10 ratio of positive to negative pairs, which means a random baseline would have an AUPR of 0.091 and an AUROC of 0.5. Bold entries represent best performance.

In Table 3, we report the area under precision recall curve (AUPR) and area under receiver operating curve (AUROC) for each model in each species. As our dataset and evaluation approach is the same as in Sledzieski *et al.* (2021), we also include results reported there for two other state-of-the-art sequence-based PPI prediction methods, PIPR (Chen *et al.*, 2019) and DeepPPI (Richoux *et al.*, 2019). We note that for unbalanced data, AUPR is generally considered the more representative metric. We also report the false positive rate (FPR) at 10% and 50% recall, which measures the likelihood that a protein pair predicted to interact is incorrectly classified—an important metric in the case where high-likelihood pairs are then tested experimentally. We find that Topsy-Turvy achieves the highest AUPR and AUROC of all the methods we evaluated in each of five species, and has the lowest FPR at both recall levels. We also observe that Topsy-Turvy retains the structural interpretability of D-SCRIPT: for each queried protein pair, the model also outputs a predicted inter-protein contact map for the putative binding between the two proteins.

*Runtime and memory usage*: Topsy-Turvy took approximately 79 h to train for 10 epochs on 421 792 training pairs, and fits within a single 32GB GPU. Running time and GPU memory usage, like in D-SCRIPT, scales quadratically, O(nm), with protein lengths *n*, *m*, since Topsy-Turvy models the full *n *×* m* contact map as an intermediate step. The prediction of new candidate pairs with a trained model is very fast, requiring on average 0.02 s/pair. Since Topsy-Turvy generalizes well across species, it needs to be trained only once on a large corpus of data and can be used to make predictions in a variety of settings. The additional run time for TT-Hybrid is minimal (approx. 15 minutes, most of it for GLIDE) since it just computes a weighted sum of predictions from Topsy-Turvy and GLIDE. The actual computation of TT-Hybrid scores, provided that the Topsy-Turvy and GLIDE results are already available, is a linear time operation (less than 1 minutes for the candidate set with 10 million pairs) since it is simply a weighted sum of the two.

#### 3.2.1 Ablation study: using network-level information for negative edge selection

Notably, Topsy-Turvy achieves greater cross-species generalization even though network information is used only during training. We hypothesize this may be partially due to GLIDE-based interaction scores mitigating the impact of incorrect labels in training data. To create negative training examples, we followed the common practice of randomly selecting protein pairs not experimentally reported as interacting (Chen *et al.*, 2019; Hashemifar *et al.*, 2018; Sledzieski *et al.*, 2021). However, it might be that such a pair actually *does* interact but has not yet been experimentally assayed. In such cases, the GLIDE score for the pair is likely to be high, thus improving the supervision and training of Topsy-Turvy. To further investigate our hypothesis, we evaluated an alternative approach to incorporating network topology in the model, by modifying the set of negative examples in the training set to reflect network information. Prior work in PPI prediction has argued that better selection of negative samples in the training set could improve the model, with Zhang *et al.* (2018) exploring a random-walk distance on the PPI graph to distinguish between and low- and high-confidence negative examples. We explored the strategy of selecting only protein pairs with low GLIDE scores as negative examples, but found the performance to be poorer than the baseline. Drilling down, we found that this was due to a reduction in diversity of negative examples available for training, since using graph-theoretic measures to select negative examples restricts us to nodes occurring in the training PPI network (Supplementary Fig. SA.1 and Appendix S1.3). In contrast, our incorporation of GLIDE scores in the objective allows us to handle a broader set of negative examples.

### 3.3 Cross-species improvement is not limited to hub nodes

Noting that Topsy-Turvy makes use of global PPI organization in the training phase but makes predictions solely using sequence data, we sought to characterize the kind of topological knowledge being learned by the trained model. Specifically, we investigated if the performance improvement of Topsy-Turvy over D-SCRIPT was limited to certain categories of proteins/nodes.

Since network-based methods work by learning network connectivity patterns, and some network structure is conserved across species, such methods tend to work well for proteins that already have many known interactions. Thus, it could be possible that the outperformance of Topsy-Turvy comes exclusively or primarily from, say, hub nodes whose interactions may be better conserved across species. To investigate this, we evaluated human-PPI trained Topsy-Turvy and D-SCRIPT on physical interactions in *D.melanogaster*, sourcing the latter from BioGRID (we found BioGRID’s fly PPI annotations clearer than STRING’s). Limiting ourselves to fly proteins that occur in the PPI network, we partitioned the fly evaluation set into four sub-groups by degree: each putative edge (*p*, *q*) was grouped as per $M_{pq}=\max(d(p),d(q))$, where *d*(*p*) and *d*(*q*) are the degrees of *p* and *q* in the fly PPI network, respectively. Thus, the sub-group corresponding to M≥21 consists of putative interactions where at least one of the proteins is a hub-like protein.

Even though baseline D-SCRIPT is not explicitly informed about network structure, it too demonstrated better performance as M increased. This may be due to the information encoded in the frequency with which each protein appears in the positive examples D-SCRIPT is trained on. Because of that, along with stronger conservation of PPIs involving hub nodes (Brown and Jurisica, 2007; Fox *et al.*, 2009), some network aspects can be implicitly learned by a purely sequence-based approach like D-SCRIPT. This also illustrates one of the core points of this article—the connection between bottom-up and top-down views of protein interaction.

We also observed that Topsy-Turvy improved upon D-SCRIPT in each sub-group, indicating that the outperformance is not only coming from high-degree nodes. While Topsy-Turvy also achieves its highest performance on the M≥21 sub-group, its improvement over D-SCRIPT is not limited to the highest-degree hub nodes. In fact, the relative AUPR improvement of Topsy-Turvy over D-SCRIPT is 2.22-fold when M is in the 2–20 range, compared to a 1.31-fold improvement for hub nodes (M≥ 21) (Table 4). Topsy-Turvy thus not only improves predictive performance for high-degree nodes, but the GLIDE loss term additionally informs the model about global structure, leading to improvement for more sparsely connected nodes.

**Table 4.** Cross-species performance of D-SCRIPT and Topsy-Turvy, subdivided by node degree in target species

Model	Overall AUPR	AUPR by maximum degree			
		2−5	6−10	11−20	≥21
D-SCRIPT	0.356	0.030	0.067	0.118	0.475
Topsy-Turvy	**0.538**	**0.073**	**0.168**	**0.237**	**0.622**

*Note*: Both methods were trained on human PPI data and tested on fly (BioGRID). The analysis is limited to protein pairs where both proteins occur in the fly PPI graph. In addition to overall AUPR, we also group each protein pair by the maximum of the degrees of its nodes in the fly PPI network. Both methods improve as maximum degree increases, and Topsy-Turvy consistently outperforms D-SCRIPT across all subsets—especially so for putative interactions between low-degree nodes. Bold entries represent best performance.

### 3.4 Topsy-Turvy’s improved performance is unlikely to be driven by ascertainment bias

In the setting where bottom-up sequence methods are compared to top-down network-based methods (or synthesis approaches like Topsy-Turvy), issues of ascertainment bias (Carter *et al.*, 2013) in the available ground truth network data become particularly acute. The issue is a simple one: existing PPI network data in all organisms [with the possible exception of recently described HuRI (Luck *et al.*, 2020)] is biased toward pairs of proteins a biologist decided to experimentally test for interaction, and biologists are more likely to include proteins already known to be of interest, or nodes that are already adjacent to other previously studied nodes in the network. The result is that nearly all ground-truth existing networks will over-estimate the performance of methods that incorporate network information, and under-estimate the performance of methods that utilize only sequence information, since missing edges are more likely to be falsely scored as negatives for the sequence-based methods. When comparing network methods against network methods, or sequence methods against sequence methods, the respective alternative is likely to be similarly biased, making it less of a concern. However, when comparing methods across both types of information, addressing the bias becomes more important.

Our results in Section 3.3 begin to address the issue of ascertainment bias. Although the BioGRID *D.melanogaster* network is not fully unbiased, if the improvement of Topsy-Turvy over D-SCRIPT were coming only from this bias, we would expect to see disproportionate improvement in the dense core of the network, where interactions are most likely to be experimentally tested. Instead, we see improvement across the network, which suggests that Topsy-Turvy’s cross-species performance gains come from successfully learning global network organization properties rather than suffering from ascertainment bias. We discuss the issue of this bias and how it might be addressed by future methods further in Discussion.

### 3.5 Comparison with AlphaFold-Multimer

We next investigated if recent advances in protein structure determination (Jumper *et al.*, 2021) that have enabled extremely high-quality protein complex structure prediction (in particular, AlphaFold-Multimer), could be leveraged for PPI prediction. While these methods were not designed to directly address *if* two proteins interact—they only predict the putative complex structure *assuming* an interaction—we investigated if AlphaFold-Multimer could nonetheless be adapted for our PPI prediction setting. From AlphaFold-Multimer results, we obtained their reported ipTM (interface predicted template modeling) score, a value between 0 and 1, that was shown in the original study to be correlated with the quality of the docked complex (DockQ score). For each candidate protein pair, we compute its mean ipTM score over the five AlphaFold-Multimer models. In our evaluations, we used this score as a predictor of protein interaction and assessed AlphaFold-Multimer on PPIs from the STRING *D.* *melanogaster* testing set used in Section 3.2.

We find that AlphaFold-Multimer is several orders of magnitude slower than Topsy-Turvy, requiring an average of 6 h per pair (AlphaFold-reported time, min = 2.87 h, mean = 5.89 h, max = 12.97 h) compared to 0.02 seconds per pair for Topsy-Turvy (hardware described in Section 2.6.1). Of the total AlphaFold-Multimer runtime, an average of 3.22 h were spent on feature generation (min = 1.62 h, max = 8.34 h) and 2.66 h were GPU time spent on model computation (min = 1.16 h, max = 4.64 h). We note that feature generation time cannot necessarily be amortized over input pairs, since an important part of adapting AlphaFold to protein complexes is the proper alignment of paired multiple sequence alignments (MSAs) for each candidate protein pair. Thus, AlphaFold-Multimer is infeasible for genome-scale *de novo* PPI prediction for organisms with limited experimental data.

We compared AlphaFold-Multimer PPI predictions with those of Topsy-Turvy in a small-scale study, constrained by the computational requirements of AlphaFold-Multimer. We selected 18 candidate pairs that span the range of Topsy-Turvy scores as well as ground-truth labels: six protein-pairs each with high (≥0.8), medium ($0.25\leq \hat{y}<0.8$) or low (≤0.25) Topsy-Turvy prediction scores, with three truly interacting and three non-interacting pairs in each subset. We note that distribution of Topsy-Turvy scores on these pairs is not representative of their full-sample distribution; for example, we expressly included examples where Topsy-Turvy was very confident but wrong, even though such instances comprise a small part of the broader distribution (89.8% of Topsy-Turvy scores are < 0.05). We found general agreement between AlphaFold-Multimer and Topsy-Turvy’s predictions (Pearson’s ρ=0.310), though there were examples where each method correctly predicted an interaction that the other missed. Full results are available in Supplementary Appendix S1.7. Compared to Topsy-Turvy, AlphaFold-Multimer’s scores seem calibrated for fewer false positives and more false negatives. In particular, AlphaFold-Multimer only scored two pairs with probability ≥0.8 both of which were true positives and also had high Topsy-Turvy scores; all other pairs were scored under 0.45. On three Topsy-Turvy false positives where it was highly confident but incorrect, AlphaFold-Multimer ipTM scores were low (mean = 0.3676). Conversely, AlphaFold-Multimer had substantial false negatives, missing three true interactions pairs that Topsy-Turvy correctly identified with medium or high probability. For pairs that Topsy-Turvy scored low, AlphaFold-Multimer agreed with it, with low ipTM scores (mean = 0.365).

These results suggest that Topsy-Turvy and AlphaFold-Multimer can each fill a valuable niche for predicting PPIs. Due to its low FPR, AlphaFold can be used to verify shortlisted interactions and accurately determine their complex structure. However, due to its run time constraints, it is infeasible to use for genome-scale predictions, a domain for which Topsy-Turvy would be more suitable. Additionally, the ipTM score is more a measure of complex stability than a predicted probability of interaction. Future work could seek to adapt the AlphaFold-Multimer architecture to explicitly address the PPI *prediction* task. For example, the calibration of interaction scores could be improved using insights gained from complete cross-docking approaches (Lopes *et al.*, 2013). Recently, Dequeker *et al.* (2022) have described physics-based energy, interface matching and protein sociability as useful metrics for identifying the likely partners from an all-versus-all docking study.

### 3.6 Integrative methods are applicable even in species with some available PPI data

We have shown that human-trained Topsy-Turvy improves on human-trained D-SCRIPT when predicting PPIs in an organism using only sequence information (Sections 3.2–3.4). In non-model organisms, there might not be any experimentally tested physical interaction data—this is the situation for which D-SCRIPT was designed, and for which we have thus far tested Topsy-Turvy. However, we are also interested in applying Topsy-Turvy to predict PPIs in the case where some sparse network does exist in the species of interest. Specifically, we ask the following question: if some network edges exist in the target species of interest, should one use a purely network-based method, or a synthesis method like Topsy-Turvy when predicting new PPIs? Sequence-based synthesis methods are necessary to attach previously unseen proteins to an existing network, but either method could be used to predict new interactions between proteins already in the network. Here, we show that a hybrid of Topsy-Turvy and GLIDE (TT-Hybrid, Section 2.5) improves upon either method alone in the case where some sparse network is available.

We consider situations where both proteins in the pair of interest occur in the PPI network, so that a network-only prediction can be made. Here, we evaluate GLIDE, Topsy-Turvy and TT-Hybrid on the *D.melanogaster* BioGRID network, which has been partitioned to measure the performance on networks of varying sparsity characterized by a parameter p∈{0.8,0.6,0.4,0.2}. More specifically, *p* describes the fraction of total edges in *G* used to construct a subset network $G_{p}=(V,E_{p})$. Full details on the construction of *G_p_* are in Supplementary Appendix S1.4. Characteristics of the sparse network datasets are described in Supplementary Appendix S1.5. The sparsified network Gp is then used to compute GLIDE scores.

To construct the test set at different *P*-values, we (a) selected the set of positive edges $S_{p}^{+}$ as all edges in *G* left out during the construction of *G_p_*, i.e. $S_{p}^{+}=E\setminus E_{p}$, and (b) randomly sampled negative examples from the set (V×V)∖E to obtain $S_{p}^{-}$. The test set $S_{p}=S_{p}^{+}\cup S_{p}^{-}$ was used to evaluate the performance of D-SCRIPT and Topsy-Turvy (trained on human), and GLIDE (trained on *G_p_*) (AUPRs in Supplementary Appendix S1.6). We also broke down the analysis into subsets of the evaluation set, based on shortest-path distance *d* in *G_p_* connecting the two proteins. Our intuition here was to check the relative performance of these methods on closely- versus distantly connected proteins. Detailed descriptions of the training network *G_p_* and the test datasets *S_p_* are provided in Supplementary Tables SA.1 and SA.2.

**Fig. 2.**
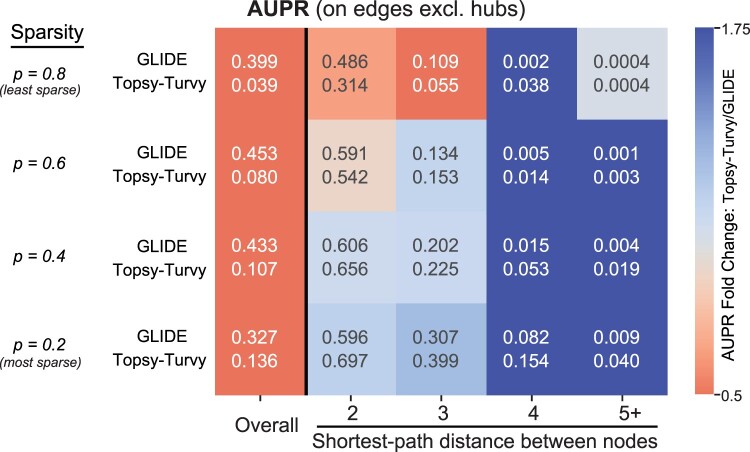
Comparing Topsy-Turvy and GLIDE in situations when both can be used. GLIDE was trained on a subset of the fly PPI network (e.g. training on 80% of PPIs when *p *=* *0.8); Topsy-Turvy was trained on human PPI data and had no access to fly data for training. Both methods were evaluated on held-out positives as well as a randomly sampled set of negative examples, where pairs containing proteins with degree ≥21 on the subset networks were removed from the held-out examples during testing; the analysis is limited to proteins in the fly PPI network. In addition to reporting overall AUPR, we also group each protein-pair in the evaluation set by their shortest-path distance in the training network

Upon initial investigation, we found that while GLIDE outperformed Topsy-Turvy overall, their relative performance on a protein pair depended on the shortest-path distance between the proteins (Supplementary Table SA.3). Since GLIDE performance is primarily driven by hubs, to more clearly investigate relative performance we then performed the same set of evaluations after removing any edges incident upon hubs [i.e. (*u*, *v*) where (degree(u)≥21)∨(degree(v)≥21)]. We then observed that Topsy-Turvy was stronger on nearly every subset of data (Fig. 2). However, GLIDE still performed better than Topsy-Turvy overall.

These results indicate that while GLIDE is able to separate PPIs by their network distance (which strongly correlates with whether or not there will be a reported interaction), once separated by network distance, Topsy-Turvy is able to finely organize similarly distant proteins using the information gleaned from sequence and structure. Thus, we introduced TT-Hybrid, which uses GLIDE and Topsy-Turvy to partition PPIs both coarsely and finely. We show in Table 5 that TT-Hybrid improves upon either component method alone, achieving the highest overall AUPR on the fly network at all levels of sparsity (with hub nodes included).

**Table 5.** TT-Hybrid improves upon both of its constituent components on in-species prediction

Sparsity	GLIDE	Topsy-Turvy	TT-Hybrid	Random
*p *=* *0.8	0.380	0.038	**0.387**	0.004
*p *=* *0.6	0.437	0.079	**0.451**	0.009
*p *=* *0.4	0.412	0.105	**0.423**	0.014
*p *=* *0.2	0.318	0.133	**0.354**	0.019

*Note*: We generated partitions of the fly network of varying sparsity, using the sparsified networks as training for GLIDE. Sparsity *p* corresponds to the proportion of edges retained in the training network (*p *=* *0.8 is the least sparse). Topsy-Turvy was trained on human PPIs. TT-Hybrid combines the predictions from both GLIDE and Topsy-Turvy. Here, we report the AUPR of each method on the held out edges removed from each network subset. We also show the AUPR of the random control; due to varying class imbalances, AUPR scores increase slightly with increasing sparsity. Bold entries represent best performance.

## 4 Discussion

We have presented Topsy-Turvy, a new method that integrates top-down global view of PPI organization into a bottom-up sequence-based PPI prediction model. The neural network design of Topsy-Turvy builds upon the architecture of D-SCRIPT and, like the latter, includes a bottleneck layer designed to model the inter-protein contact map, thus offering interpretability and insight into the mechanism of interaction. We show that Topsy-Turvy is highly accurate in a cross-species context, and applicable to species with few or no known protein interactions. For cases where PPI data is available in the target species, we present TT-Hybrid, that can leverage this additional information for more accurate predictions.

Topsy-Turvy thus improves upon the state-of-the-art in PPI prediction broadly—both in species without available PPI data and in those with PPI data. For the former, it is able to transfer knowledge of network structure from other species, leading to more accurate *de novo* predictions. For the latter, it improves prediction coverage as well as accuracy. For instance, even in well-studied species like human, mouse, and fly, there remain many proteins with no characterized PPIs [24.9%, 44.9% and 19.8% of proteins in the three species, respectively (Pray, 2008; Serres *et al.*, 2001)]. Topsy-Turvy can be used to attach these hitherto uncharacterized proteins to existing PPI networks. Since GLIDE and other network methods are limited to predicting links between proteins that both already exist in the network, they cannot be used for putative interactions involving such proteins. When both proteins do exist in the PPI network, the hybrid approach TT-Hybrid that combines GLIDE with Topsy-Turvy performs better than either approach alone, with the former achieving a coarsely accurate network-theoretic organization and latter fine-tuning it locally. Here, we hypothesize that GLIDE confers species-specific network information unable to be transferred by Topsy-Turvy due to network rewiring.

The TT-Hybrid results also give some hint as to what Topsy-Turvy might be learning from including a network loss term in the *training* stage. As shown in Figure 2, the GLIDE network score helps segregate proteins into buckets that give a macro range of potential probabilities that an edge exists, while the bottom-up sequence approach does best at ranking the specific pairs within each bucket. This is not the first time we have seen network-based information assist in making sequence-level information more accurate; the Isorank network alignment algorithm (Singh *et al.*, 2007) also receives a gain in performance in discovering orthologs by a global top-down network similarity score that augments the bottom-up pairwise sequence score.

In this regard, Topsy-Turvy presents an approach to an often-faced challenge in systems biology: how to resolve the dichotomy between a bottom-up and top-down view of the same biological phenomenon? Considered at the molecular level, protein interaction is a purely physicochemical process. However, these proteins primarily function through their interactions. With proteins performing most of the functions in the cell, evolution constrains the space of possible protein folds, resulting in emergent properties at the network level. The approach embodied by Topsy-Turvy and TT-Hybrid could be more generally applied to situations where network-theoretic and molecular views need to be integrated. To make a social interaction analogy, D-SCRIPT and other sequence-based bottom-up methods are learning features that make two people likely to be compatible as friends, but not global organization of the friend network that would indicate if those two people share enough mutual friends to be likely to have had the opportunity to meet at the same event.

While we took steps to rule out the effect of ascertainment bias, this remains an important question in both the training and evaluation of link prediction methods. In this work, we sourced PPIs from the STRING database where data from a variety of assays has been conglomerated. An unbiased, all-versus-all screen as exemplified by the Human Reference Interactome (HuRI) database (Luck *et al.*, 2020) offers the promise of addressing ascertainment bias in the specific case of yeast two-hybrid (Y2H) screens. However, to test Topsy-Turvy in our transfer-learning context, we would also need similar unbiased Y2H screens in a different species.

By approaching integration of orthogonal information sources as a multi-objective learning problem, Topsy-Turvy lays the groundwork for incorporation of additional data modalities. For instance, while the GLIDE score incorporates both global and local scores, it would be possible to directly supervise Topsy-Turvy with global and local loss terms, each with a respective hyper-parameter to finely control their effects. Loss terms that quantify protein functional similarity (Ghersi and Singh, 2014) or interface similarity (Budowski-Tal *et al.*, 2018; Gainza *et al.*, 2020) could be added to the framework to further inform predictions. Topsy-Turvy demonstrates that a general, scalable framework that allows us to transfer both low-level (sequence-to-structure) and high-level (network topology) insights across species can enable researchers to fill in the missing links in our knowledge of biological function.

## Funding

R.S., S.S. and B.B. were supported by the National Institutes of Health grant [R35GM141861]. K.D. and L.C. were supported by National Science Foundation (NSF) grant [CCF-1934553]. S.S. was supported by the National Science Foundation Graduate Research Fellowship [1745302].

*Conflict of Interest*: none declared.

## Supplementary Material

btac258_Supplementary_Data
