# Supplementary material for: Topsy-Turvy: integrating a global view into sequence-based PPI prediction
Source: Bioinformatics. 2022 Jun 27;38(Suppl 1):i264–72. doi: 10.1093/bioinformatics/btac258 (PMC9235477; doi:10.1093/bioinformatics/btac258)
Supplement: btac258_Supplementary_Data [file btac258_supplementary_data.pdf]

1 Appendix

1.1 Description of Local and Global similarity scores used in GLIDE

Local similarity score: Common Weighted Normalized Given nodes $p, q \in G$, the Common Weighted Normalized (CWN) score is

$$\mathrm{CWN}(p,q) = \frac{\sum_{r \in \mathcal{N}_p \cap \mathcal{N}_q} (w_{p,r} + w_{q,r})}{\sqrt{k(p)k(q)}}$$

where for any node $x \in G, \mathcal{N}_x$ is the neighbor set of x, $w_{x,y}$ is the weight of the edge (x,y) and $k(x)$ represents the weighted degree of x. Note that this is slightly different from the CW metric described in (Devkota *et al.*, 2020), because of the square roots in the denominator, which we found corrected an overweight on the interactions between high-degree hub nodes from the original CWN used in GLIDE, improving performance.

Global similarity score: $\mathbf{UDSED}^\gamma$ *Distance* We first describe the DSE^γ embedding that forms the basis of this scoring scheme (from (Devkota *et al.*, 2020)). Let P be the Markov transition matrix computed from a graph G with the unique stationary distribution π and let D be the diagonal degree matrix representing the weighted degree of all the nodes in the network. Then the DSE^γ embedding is:

$$DSE^\gamma = I + \sum_{t=1}^{\infty} \gamma^t (P - W)^t, \tag{5}$$

where W is a constant matrix, whose rows are copies of the stationary distribution π and γ is a parameter satisfying $0 < \gamma \leq 1$, which is used to control the contribution of larger time-steps in the computation of the embedding. We set $\gamma = 1$ in all our experiments, as suggested in (Devkota *et al.*, 2020).

If $\mathrm{DSE}^\gamma(p)$ and $\mathrm{DSE}^\gamma(q)$ represent the DSE^γ embeddings for the nodes p and q respectively, we consider the un-normalized L2 distance between their DSE^γ embeddings. Again, this is a variation from *normalized* L2 distance described in (Devkota *et al.*, 2020). Formally, this can be written as

$$\mathrm{UDSED}^\gamma(p,q) = \sqrt{\sum_k (\mathrm{DSE}^\gamma(p)_k - \mathrm{DSE}^\gamma(q)_k)^2} \tag{6}$$

1.2 Data set generation

In order to select only high-confidence physical protein interactions, we limited our positive examples to binding interactions associated with a positive experimental-evidence score. From this set, we removed PPIs involving very short proteins (shorter than 50 amino acids) and, due to GPU memory constraints, also excluded proteins longer than 800 amino acids. Next, we removed PPIs with high sequence redundancy to other PPIs Specifically, we clustered proteins at the 40% similarity threshold using CD-HIT , and a PPI (A-B) was considered sequence redundant (and excluded) if we had already selected another PPI (C-D) such that the protein pairs (A, C) and (B, D) each shared a CD-HIT cluster. Removing sequence redundant PPIs from the data set prevents the model from memorizing interactions based on sequence similarity alone (Sledzieski *et al.*, 2021).

1.3 Considering network structure for negative sample selection has marginal impact

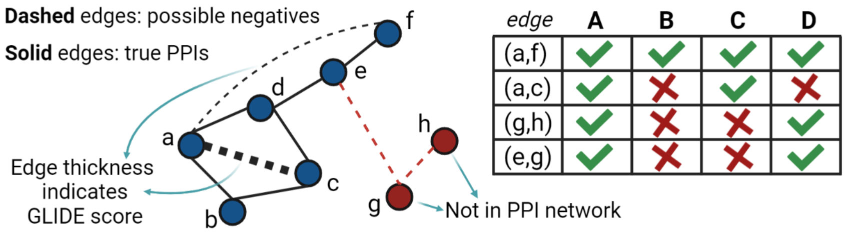

	Negative Sampling Method	AUPR	AUROC
**A**	All nodes; random pairs	0.605	0.890
**B**	PPI nodes only; weak GLIDE scores	0.380	0.840
**C**	PPI nodes only; random pairs	0.477	0.832
**D**	All nodes; exclude strong GLIDE scores	**0.629**	**0.913**

Figure A.1: Using network information to guide selection of negative training examples. The common practice in PPI prediction literature is to consider random pairs of proteins as negative examples (row **A**). Restricting negative examples to just low-scoring GLIDE node pairs (row **B**) results in substantially weaker performance, likely due to reduced diversity of negative examples. Ablation studies (rows **C** and **D**) support the hypothesis. AUPR and AUROC were computed using a D-SCRIPT model trained on human protein interactions, with negative edges generated by each respective method, and evaluated on the *D. melanogaster* STRING network.

We assessed if using GLIDE scores to select negative training examples results in higher quality training data and improved model performance. We first applied GLIDE on the PPI network formed by positive examples in the training set, calculating a GLIDE score for all pairwise combinations of nodes (i.e. proteins) in this network. The negative examples were then chosen from these as the k lowest-scoring protein pairs; as in the previous experiments, we chose k to ensure a 10:1 negative-to-positive ratio. On this modified training set, we trained a vanilla D-SCRIPT model (i.e., no network loss in the objective) on human PPI data and evaluated it on *D. melanogaster* interactions.

Surprisingly, the baseline version of D-SCRIPT (trained on negative examples chosen completely at random) had substantially stronger performance than the version of D-SCRIPT trained on a network-informed training set (rows A vs. B in Figure A.1). We wondered if the latter's lower performance was due to a reduced diversity of negative

examples in the latter: using GLIDE to select negative examples limits us to protein pairs where both proteins exist in the PPI network. Since experimental PPI data is limited, the negative examples are restricted to only a subset of the human proteome. In contrast, the baseline version of D-SCRIPT includes negative examples where one or both the proteins might not occur in the positive examples. To test our hypothesis, we trained and evaluated two other variants: i) choose negative examples completely at random, but limit the proteins to those occurring in the PPI network, and ii) in the baseline training set, remove all negative examples (p, q) where p and q both occur in the PPI network and replace them with an equal number of examples (p', q') where p' and q' also occur in the PPI network but (p', q') has a low GLIDE score. As we hypothesized, the first variant (row C in Figure A.1) performed worse than the baseline D-SCRIPT (row A). The second variant (row D) performed marginally better than the baseline, suggesting that the incorporation of network information in the training set construction does help somewhat. However, this improvement was marginal and unclear (for instance, row C has a lower AUROC than row B), so we chose not to incorporate it into Topsy-Turvy.

1.4 Maintain minimum spanning tree while sparsifying network

Consider a graph $G = (V, E)$, which we use to generate a sparsified sub-graph $G_p = (V, E_p)$ using a parameter p, which denotes the fraction of G's edges retained in G_p. We require G_p to have same connectivity as G because graph connectivity is required for many network-based link prediction methods. To ensure that G_p is connected and all the nodes in G are included in G_p, we perform the following operations, following (Devkota *et al.*, 2020).

1. Compute a random spanning tree ($T = (V, E_T)$) from G, where $|E_T| = |V| - 1$ (We used Kruskal's algorithm (Kruskal, 1956) for this, whose computational complexity is $|E| \log |V|$).
2. Let $S = E \setminus E_T$. Randomly add $p|E| - |E_T|$ edges from S to the constructed tree T to produce $G_p = (V, E_p)$.

The remaining edges in S, that were not added to construct G_p, were then used as the positive examples for experiments in Section 3.

1.5 Sparsity data set characteristics

Here we provide information about the data sets generated in the sparsified fly network analysis (Tables A.1, A.2).

Table A.1. Network Information of sparsified fly network G_p for different p-values

Sparsity	#Nodes	#Edges	Diameter	Average Degree
$p = 1.0$	3093	27134	17	17.54
$p = 0.8$	3093	21707	18	14.03
$p = 0.6$	3093	16280	17	10.52
$p = 0.4$	3093	10853	18	7.01
$p = 0.2$	3093	5426	22	3.50

Table A.2. Positive and Negative test examples for different p and k values

**Sparsity**	**Data Set**	**Overall**	**By shortest path bin**			
			2	3	4	5+
$p = 0.8$	Positive	5085	4824	211	36	14
	Negative	267173	8841	32942	63243	162147
$p = 0.6$	Positive	10183	9314	696	100	73
	Negative	267173	6809	26647	56987	176820
$p = 0.4$	Positive	15287	12957	1883	261	186
	Negative	267173	4652	21768	50164	190589
$p = 0.2$	Positive	20352	12343	5661	1378	970
	Negative	267173	2418	13985	35915	214855

1.6 Effect of shortest path in training network (including D-SCRIPT)

In order to illustrate the deviation in performance for predictions with different graph distances in more detail, we devise the following experiment, using graph of various sparsity:

1. For a given p, compute G_p and S_p described in Section 3.6.
2. For a given k, find the node-pairs in S_p having the shortest graph distance, the graph being G_p, equal to k. Call this set $R_{p,k}$.
3. Train GLIDE on the graph G_p, and compute the resulting GLIDE scores for node-pairs in $R_{p,k}$.
4. Compute scores and metrics for node-pairs in $R_{p,k}$.

This experiment was done for $k \in \{2, 3, 4, 5\}$ and $p \in \{0.8, 0.6, 0.4, 0.2\}$. Table A.3 demonstrates the corresponding AUPR scores.

One of the reasons behind the difference in AUPR results between GLIDE and D-SCRIPT/Topsy-Turvy is the significant advantage GLIDE has in predicting links between node-pairs that are very close to each other in the PPI network. As Topsy-Turvy and D-SCRIPT are not trained on any network characteristics specific to the target organism, we observe it lagging behind GLIDE in overall performance (Table A.3). However, there do appear to be regions of the network where Topsy-Turvy and D-SCRIPT perform better than GLIDE.

GLIDE is shown to be very effective in correctly predicting interaction in the core regions of the network where majority of the hub proteins and their corresponding interactions reside. On the other hand, it is far more challenging to predict interactions between proteins in the peripheral region of the PPI network, where the interactions are largely unexplored. To see if sequence-based methods like D-SCRIPT and Topsy-Turvy perform better in this region, we construct the following experiment:

1. Generate a set of hub nodes H from the complete network G, by selecting G's nodes having degree above a certain cutoff d_c.
2. Given p, construct G_p and S_p as above. Train GLIDE on G_p.
3. Filter out protein-pairs from S_p if either of the protein is contained in H to produce S'_p.
4. For a given k, find the protein-pairs in S'_p having the shortest graph distance, the graph being G_p, equal to k. Call this set $R_{p,k}$.
5. Compute scores and metrics fo rthe pairs in $R_{p,k}$.

We report AUPR scores for D-SCRIPT, GLIDE and Topsy-Turvy on the hub-free data sets in Table A.4.

Table A.3. AUPR scores for D-SCRIPT, Topsy-Turvy, and GLIDE, for different values of k and p, including hub nodes

Sparsity	Model	Overall AUPR	AUPR by Shortest Path 2	3	4	5+
$p = 0.8$	GLIDE	**0.8057**	**0.8370**	**0.1186**	0.0016	0.0003
	D-SCRIPT	0.1256	0.5169	0.0184	**0.0039**	**0.0007**
	Topsy-Turvy	0.2442	0.5506	0.0260	0.0007	0.0002
$p = 0.6$	GLIDE	**0.8398**	**0.8847**	**0.1379**	0.0041	0.0009
	D-SCRIPT	0.2051	0.7159	0.0583	0.0060	0.0009
	Topsy-Turvy	0.3668	0.7412	0.0781	**0.0072**	**0.0019**
$p = 0.4$	GLIDE	**0.8180**	**0.8763**	**0.2612**	0.0111	0.0035
	D-SCRIPT	0.2762	0.8376	0.1385	0.0234	0.0037
	Topsy-Turvy	0.4529	0.8529	0.1846	**0.0253**	**0.0112**
$p = 0.2$	GLIDE	**0.7379**	0.8256	**0.6702**	**0.1337**	0.0112
	D-SCRIPT	0.3277	0.9161	0.4836	0.0734	0.0123
	Topsy-Turvy	0.5095	**0.9224**	0.5430	0.1171	**0.0311**

Table A.4. AUPR scores for D-SCRIPT, Topsy-Turvy, and GLIDE, for different values of k and p, after the removal of hub nodes.

Sparsity	Model	Overall AUPR	AUPR by Shortest Path 2	3	4	5+
$p = 0.8$	GLIDE	**0.3993**	**0.4857**	0.1094	0.0022	0.0004
	D-SCRIPT	0.0143	0.2509	0.0365	0.0082	**0.0015**
	Topsy-Turvy	0.0389	0.3141	**0.0545**	**0.0375**	0.0004
$p = 0.6$	GLIDE	**0.4535**	**0.5910**	0.1336	0.0054	0.0011
	D-SCRIPT	0.0280	0.4436	0.1035	0.0114	0.0015
	Topsy-Turvy	0.0804	0.5422	**0.1528**	**0.0142**	**0.0031**
$p = 0.4$	GLIDE	**0.4329**	0.6055	0.2022	0.0150	0.0037
	D-SCRIPT	0.0398	0.5624	0.1760	**0.0395**	0.0063
	Topsy-Turvy	0.1067	**0.6562**	**0.2245**	0.0150	**0.0192**
$p = 0.2$	GLIDE	**0.3274**	0.5956	0.3068	0.0822	0.0094
	D-SCRIPT	0.0521	0.6112	0.3033	0.0910	0.0175
	Topsy-Turvy	0.1359	**0.6970**	**0.3986**	**0.1539**	**0.0399**

1.7 Comparison with AlphaFold-Multimer

We evaluated Topsy-Turvy and AlphaFold-Multimer on 18 protein pairs from the *D. melanogaster* STRING network. Due to runtime constraints for Alphafold-Multimer, we attempted to cover a diverse set of pairs which span a wide range of Topsy-Turvy scores and including both positive and negative interactions.

Table A.5. We report the predicted probability of interaction from Topsy-Turvy and AlphaFold-Multimer (Mean ipTM over 5 models) for 18 candidate fly protein pairs. We also report the full run time of AlphaFold-Multimer in seconds, as well as the times for HMM search and feature generation (Feature Time) and total prediction time over five models (GPU Time). Each Topsy-Turvy prediction takes approximately 0.02 seconds.

ID1	ID2	Interaction Label	Topsy-Turvy Score	Mean ipTM	Full Time (s)	Feature Time (s)	GPU Time (s)
7227.FBpp0078386	7227.FBpp0112233	0	0.8639	0.2453	24714.6914	17229.8331	7484.8582
7227.FBpp0071140	7227.FBpp0297773	0	0.8824	0.2152	22462.4294	6670.0248	15792.4046
7227.FBpp0072955	7227.FBpp0080316	0	0.9715	0.3169	19735.4847	8131.6765	11603.8082
7227.FBpp0088242	7227.FBpp0088439	1	0.8319	0.3660	31796.5017	24201.2336	7595.2680
7227.FBpp0085619	7227.FBpp0086223	1	0.8383	0.8059	11169.2248	6975.8700	4193.3548
7227.FBpp0113041	7227.FBpp0290012	1	0.8270	0.8503	13510.7173	6942.3823	6568.3350
7227.FBpp0080022	7227.FBpp0303413	0	0.0004	0.3939	18286.9680	6986.6605	11300.3075
7227.FBpp0078625	7227.FBpp0086780	0	0.0001	0.3786	21367.1549	10417.7165	10949.4383
7227.FBpp0071149	7227.FBpp0074705	0	0.0003	0.1734	22037.3726	6207.0115	15830.361
7227.FBpp0071136	7227.FBpp0088946	1	0.0004	0.2895	20386.5746	10035.5682	10351.0064
7227.FBpp0079704	7227.FBpp0304061	1	0.0102	0.2173	19813.0249	10494.3563	9318.6685
7227.FBpp0085164	7227.FBpp0087553	1	0.0026	0.3683	20318.7372	7338.5067	12980.2304
7227.FBpp0072055	7227.FBpp0073836	0	0.3921	0.3157	46704.2127	30016.3854	16687.8272
7227.FBpp0083802	7227.FBpp0087849	0	0.4864	0.2269	19511.2074	13965.8906	5545.3167
7227.FBpp0073028	7227.FBpp0086215	0	0.7401	0.2924	13408.1489	7660.8085	5747.3404
7227.FBpp0071046	7227.FBpp0079780	1	0.6129	0.3442	33131.6638	22314.3092	10817.3545
7227.FBpp0082370	7227.FBpp0100175	1	0.2989	0.3197	12807.1603	7447.1986	5359.9617
7227.FBpp0076184	7227.FBpp0079616	1	0.4962	0.4495	10344.6763	5836.6977	4507.9785
